# Effect of play-based intervention on children’s mental status and caregiver involvement during hospitalization: findings from Pakistan

**DOI:** 10.1186/s12887-024-04659-5

**Published:** 2024-04-04

**Authors:** Vardah Noor Ahmed Bharuchi, Muneera A. Rasheed

**Affiliations:** 1 Department of Paediatrics and Child Health, The Aga Khan University, National Stadium Road, Karachi, Pakistan; 2 Centre for International Health, Department of Global Public Health and Primary Care, University of Bergen, Bergen, Norway

**Keywords:** Hospitalization, Children, Parent mediated therapy, Play stimulation, Parent-child relationship, Pretest-posttest

## Abstract

**Background:**

The nurturing care framework (NCF) encompasses responsive caregiving, health, nutrition, safety and security by parents and other caregivers. It improves health, development and wellbeing of children. A hospital environment can be detrimental to the developmental and emotional needs of children hence NCF can be applied to hospitalized children.

**Objective:**

The objective was to determine if (i) play stimulation intervention mediated by non-specialist providers (caregivers) improves mental status of children who are hospitalized; (ii) to examine if difference varies between different providers and iii) if there is variation based on child age and criticalness of illness.

**Method:**

A one-group pretest-posttest research was carried out using purposive sampling in a pediatric unit in Karachi, Pakistan, from November 2017 to December 2019. Children aged 3 months to 6 years were offered play stimulation by trainee psychologists. The outcome was measured through an observation tool, the Mental Status Examination Scale (MSE-S) developed for the study.

**Results:**

A total of 524 sessions were delivered to 351 children. Significant mean difference was observed on MSE-S before and after the intervention when it was provided by trainees (9.95, CI = 8.11, 11.7), mothers (mean difference = 5.86, CI = 5.30, 6.42), fathers (mean difference = 5.86, CI = 4.48, 7.24) and non-specialist providers [caregivers (mean difference = 5.40, CI = 3.91, 6.89). Significant differences in mean was observed on MSE-S across different age groups and criticalness of illness.

**Conclusion:**

It was concluded that play stimulation not only affects the behaviour of children but also varies when delivered by caregivers and trainees. Hence, interventions that involve parents are feasible.

**Supplementary Information:**

The online version contains supplementary material available at 10.1186/s12887-024-04659-5.

## Background

Hospitalization can be a stressful experience for both children and adults, impacting mood, behavior, cognitive function, and the parent-child relationship [1]. This stress manifests in changes to a child’s mental state, encompassing factors like concentration, memory, orientation, appearance, and judgment [2, 3]. Research also shows increased anxiety, pain, and fatigue in hospitalized children compared to before hospitalization [4, 5]. Additionally, conditions like congenital heart disease, intestinal failure, and malnutrition can affect cognitive and motor development, while chronic illness can lead to a lower quality of life [6–10].

Interventions exist to address these challenges, with play stimulation offering a promising approach. Engaging in play helps children adapt to stress, fosters cognitive, social, emotional, and motor development, ultimately contributing to better health outcomes [11]. Simple interventions like daily coloring or pretend play have demonstrated effectiveness in reducing anxiety and improving cognitive function in hospitalized children [4, 12].

These interventions can be provided by parents if they are taught to them. Play-based interventions provided to parents can influence both parent and child behavior. Emotional cues like facial expressions, voice tone, and posture play a crucial role in parent-child interactions, and psychosocial interventions for parents can lead to improved psychological functioning for both [13, 14]. Moreover, creating a cognitively stimulating home environment after critical illness or procedures, facilitated by parents or caregivers, can further enhance children’s cognitive development [15]. Involving parents in psychosocial care during hospitalization can provide additional stimulation and support for children to be continued at home.

However, psychosocial interventions are often delivered by specialists trained in child life. These professionals work to minimize the adverse effects of hospitalization, teach coping skills, and involve parents in the healthcare process [1]. Recognizing the need for such specialists, multidisciplinary teams have emphasized the importance of play-trained personnel for both children and their parents [16]. This need was further highlighted during the pandemic, where the value of mental health specialists in supporting patients became evident [17].

While the detrimental effects of hospitalization on children’s mental health are well-documented globally, the challenge takes on a unique dimension in Pakistan. Limited resources in Pakistani hospitals pose a significant barrier to readily available psychosocial interventions delivered by trainees, leaving a glaring gap in addressing the mental health needs of hospitalized children [18]. This gap not only affects immediate emotional well-being but can also potentially impede long-term development and quality of life [5–9].

Recognizing this critical need, the present study seeks to bridge the gap by investigating the effectiveness of a novel play-based intervention. This approach harnesses the established benefits of play in mitigating stress, fostering development, and improving mental state in children [10–12]. Moreover, the intervention prioritized parental engagement, acknowledging the crucial role parents play in their children’s emotional well-being and the potential benefits of parent-mediated psychosocial interventions [13–15]. The intervention is inspired by the aforementioned principles and the nurturing care framework for early child development [19, 20].

Recognizing the limited human resources as a challenge, the current study seeks to address the gap by implementing intervention via non-specialists as task-shifting approach. Building on this foundation, the present study has three key objectives: (i) to determine if a play stimulation intervention delivered by non-specialist providers can improve the mental state of hospitalized children; (ii) to examine whether sessions mediated by parents differ from those mediated by other providers in their impact on the child’s mental state before and after the intervention; iii) to examine if there was variation in the difference by child age and type of care (acute, special and critical).

## Methods

### Study setting

This is an implementation research of play stimulation intervention that was carried out in the paediatric ward of a private hospital in Karachi, Pakistan using purposive sampling from November 2017 to December 2019. The paediatric ward has two units that comprises of 84 beds in which five beds area is for neurology, 24 cots in advanced neonatal intensive care unit and paediatrics intensive care unit. People from all over Pakistan and neighbouring countries like Afghanistan, Iran and Iraq visit for treatment and invasive medical procedures. The hospital does not adhere to people from one specific socio-economic class, but to people from all socio-economic backgrounds. Ethical approval was sought from the institute’s Ethical Research Committee for the study.

### Implementation strategy for the intervention

In the first phase a theory of change model was created with the final outcome of reduced stress in children and their families during hospitalization. Internal and external resources were identified. Based on the final outcome, a package was created which consisted of the intervention manual of children aged one month to six years with activities pertaining to cognitive, socio-emotional, language and fine and gross motor skills. The package also had behavioral observation forms of parents and children as well as the mental status examination form for children. Parental feedback was also taken in the last session. Training manuals for nurses and trainee psychology students were also created. Students’ performance was marked on the supervision observation checklist that assessed the student’s skills on active listening, core therapeutic conditions and the interventions they provided. Summary of the intervention package is given in Table 1. Details have been part of another manuscript [19].

**Table 1:** Summary of the intervention package

Domain	Type	Brief Description	Population
**Intervention Package/Materials**			
Play Stimulation Package	Intervention manual	Empirical based play activities on physical, language, cognitive and psychosocial development for different age groups.	Families of children aged newborn to six years
Training Package for Therapists	Training	Child development, importance of play in hospital, roles and responsibilities of therapists, principles of the program, and introduction of group and individual play stimulation programs. Brief guideline prompts were also created.	Therapists
Training Package for Nurses	Training	Brief training included all the aspects from the training package for nurses. The training was for two days.	Nurses
Infection Control Standard Operating Procedure (SOP)	Infection Control	Guidelines on ways to disinfect and sanitize the toys based on the use and type of disease. It also consisted of the checklist that trainees had to use before and after using the toys.	Trainees
**Exposure to the Intervention**			
Group sessions	Intervention	Maximum five to six participants from different age groups. Children younger than 2 years to receive interventions from manual for 40 min children older than 2 years for fifty minutes.	Children aged newborn to six years, parents and trainees.
Individual sessions	Intervention	Play interventions from the manual on the bedside for parents and children between 20 to 50 min.	Children aged newborn to six years, parents and trainees.
**Training**			
Training	Training	Trainees who were enrolled in post-graduate psychology programs were hired. They were given training from the training package of nurses. Training was for two days with observations.	Trainees
Supervision	Supervision	The session was held once a week in which cases were discussed, grievances and feedback from the observation checklist were shared.	Trainees and investigators
**Intervention Implementation Measures**			
Mental Status Examination	Instrument	Demographic information and before and after intervention observation on motor skills, communication, mood, attention and multiple choices on theme of play and transition out of session. The scale was completed by trainee before and after intervention.	Children
Individual Family Behaviour Checklist	Instrument	Likert-type scale observational tool that looks into parental behaviour with children during hospitalization. The scale was completed by the trainee after intervention.	Parents, grandparents, uncles and aunts.
Individual Behaviour Checklist	Instrument	Likert type scale observation tool that looks at the behaviour of children when hospitalised. The scale was completed by the trainee after intervention.	Children
Group Session Follow-up Form	Observation Form	Goals of therapy, observation based on multiple choice on the themes of play, pro-social behaviour and transition out of session. This form was to be completed by the trainees after the session.	Children
Family Feedback Form	Survey	Likert type scale with seven questions that constituted satisfaction with the program, challenges faced and impact of intervention increasing positive behaviour. This form was to be completed by the parents in the last session.	Parents
Quarterly Meeting Record Sheet	Record form	Intervention progress, objectives and solutions to problems. This form was to be completed by the investigators.	Investigators
Supervision Observation Checklist	Observation Form	Dichotomous questions under the domains of organization, structure of the session, core conditions, boundary setting, interaction with individuals, cooperation and strength and weaknesses. This form was completed by the investigators.	Trainees

### Data collection measures

#### Mental status examination scale

Mental Status Examination Scale (MSE-S) form included demographic information. Some segments of the form include the observation before and after the session such as motor skills, communication, mood and attention. Some items on the scale were measured via multiple choices such as transition out of session and theme of play [21]. Some questions were dichotomous such as the orientation of person place and time and some questions were multiple response questions such as for mood and transition out of session [21]. The total score was calculated out of 60. The domains were general appearance, motor skills, speech, communication, mood and affect, orientation, thought content, insight and judgement and type of interaction with caregivers and therapists. The scale is a combination of questions that require rating and branching scales [22]. A detailed description of the scale can be found in Bharuchi & Rasheed, 2021 [23].

### Data collection procedures, data management and analysis

The study pertains to children who were offered therapy. Since the resources for providing interventions were limited, all the children within the ward could not receive intervention. Some of the children were offered therapy on the request of the consultants. Some children were identified by the trainees on bedside and consent was sought from caregivers, nurses and residents. Children were identified based on their age as well as whether they were sleeping and if they had any upcoming procedures. Hence, purposive sampling was used for data collection. Sample size was not calculated as this was a quality improvement initiative [24].

The data was collected before and after the intervention by trainees while the caregivers conducted the session. For some sessions the trainees offered the session by taking the permission from bedside nurses or the residents in the ward as the caregivers would be with the consultants or would have left for breakfast. Children were observed in terms of their movements, interaction with their caregivers as well as the play that they were engaged in before and during the intervention. Each intervention session duration was approximately 20 to 40 min, depending on the age of the child. The younger the child, the less the duration of the session. The interventions were shared with caregivers, and they were instructed to do the activities with children. Based on the interaction as well as children’s own behaviour and response to the caregiver and play, children were marked on the MSE-S. The data from the MSE-S was collected via Google Forms. The data was then compiled on Microsoft Office Excel software where it was cleaned and diseases and names were rechecked with the discharge summaries submitted to the hospital’s online portal of patients and then coded into numerical values by the first author.

Descriptive statistics was used to summarize quantitative data. Frequencies were used for the summation of quantitative data on IBM SPSS. Paired samples T test was used to check the difference between the overall scores of mental status examination before and after the intervention as well as age, caregiver relationship and criticalness of illness (acute care, special care and critical care). Paired Samples T test was also used to check the difference in the scores of MSE-S before and after intervention in acute care when the intervention was given by parents, caregivers, and trainees. This test was specifically carried out due to the findings of behavioral observation of parents and children. The independent samples t test analysis of behavioral observation is given in supplementary file. Behavioral observation comprised of communication between the caregiver and child as well as responsiveness of the parent. It included the child and the caregiver’s stress, positive affect and negative affect based on the tone, facial expression and body language.

## Results

Table 2 shows the frequency of the demographic variables of the study. Children of different diseases participated in the program. 36.5% of the children in the study were 4 to 6 years old. There were more males (59.2%) than females (40.7%). The average age of mothers was 30.5 years, whereas the average age of fathers was 35.8 years. Most of the participants received interventions in the acute care area (74.5%). 66.9% of the children received 1 session and 19.6% of the children received 2 sessions. Some children received more than 3 sessions. Around 68.57% of the time the mothers were on the bedside followed by fathers (14.9%).

**Table 2:** Frequency of Sociodemographic Variables

Variable	N (%)
**Age**	
0–6 months	44 (12.5)
7–12 months	54 (15.9)
13–24 months	45 (12.8)
25–36 months	80 (22.8)
36–60 months	128 (36.5)
**Gender**	
Male (N,%)	208 (59.2)
Female (N, %)	143 (40.7)
**Diseases**	
Cardiovascular Diseases	98 (27.9)
Infectious Diseases	51 (14.5)
Respiratory Diseases	46 (13.1)
Gastroenterological Disorders	29 (8.3)
Cancer	20 (5.7)
Nephrotic Disorders	20 (5.7)
Neurological Disorders	17 (4.8)
Orthopedic Disorder	10 (2.9)
Others	35 (10.0)
**Type of care**	
Critical care	47 (9.0)
Special care	75 (14.3)
Acute care	391 (74.5)
**No. of sessions**	
1	351 (66.9)
2	103 (19.6)
3	30 (5.7)
4	15 (2.9)
5–12	25 (4.78)
**Attendant present during the session**	
Mother	360 (68.57)
Father	78 (14.86)
Others	46 (8.76)
No caregiver	41 (7.81)
**Age of parents (Mean, SD)**	
Mother (212)	30.54 (5.8)
Father (209)	35.8 (7.0)

Note: age, gender and age of parents have been calculated based on session 1 since they remained constant in other sessions. Type of care and attendant present during the session are calculated from all the sessions combined. From the age of parents, data of 142 fathers is missing and of mothers, data of 139 mothers was missing

Table 3 shows the results of Paired samples T test. It shows an overall significant difference in the scores of MSE-S before and after the intervention (mean difference = 6.14; t(524)=-25.04, *p* < 0.001). When examined by providers, greatest difference was observed on MSE-S before and after the intervention when offered by trainees (mean difference = 9.95; t [38] = 10.91, *p* < 0.001), mothers (mean difference = 5.86; t(360) = 20.57, *p* < 0.001) and fathers (mean difference = 5.86; t (77) = 8.54, *p* = < 0.001) but not by other caregivers.

Significant differences before and after intervention on MSE-S were also observed across different age groups such as 13–24 months mean difference = 6.06; t(75) = 8.30,=*p* < 0.001). The table also shows a significant difference on MSE-S in special care before and after intervention (mean difference = 6.41;, t(74) = 10.66, *p* < 0.001).

**Table 3:** Paired Samples T test of MSE before and after intervention based on the presence of attendants, age and type of care

Domain	N	Before Mean (SD)	After Mean (SD)	Mean Difference (95% CI = LL, UL)	*t*	df	Sig
Total MSE	525	35.92 (10.42)	42.06 (10.44)	6.14 (5.56, 6.62)	25.04	524	< 0.001
**Attendant present during the session**							
Mother	361	35.55 (10.29)	41.41 (10.47)	5.86 (5.30, 6.42)	20.57	360	< 0.001
Father	78	36.22 (11.88)	42.08 (11.38)	5.86 (4.48, 7.24)	8.45	77	< 0.001
Other caregivers (siblings, grandparents, uncles and aunts)	45	38.69 (9.38)	44.09 (8.54)	5.40 (3.91, 6.89)	7.30	44	< 0.001
Trainees	41	35.61 (9.46)	45.56 (9.59)	9.95 (8.11, 11.7)	10.91	40	< 0.001
**Age**							
0–6 months	60	31.38 (11.43)	37.13(10.63)	5.75 (4.32, 7.27)	7.58	59	< 0.001
7–12 months	78	32.21 (10.71)	38.27 (9.78)	6.06 (4.95, 7.18)	10.81	77	< 0.001
13–24 months	76	35.91 (10.15)	41.92 (8.87)	6.06 (4.56, 7.46)	8.30	75	< 0.0010
25–36 months	112	36.21 (9.98)	43.23 (11.05)	7.02 (5.89, 8.14)	12.35	111	< 0.001
36–60 months	199	38.59 (9.48)	44.44(10.01)	5.85 (5.11, 6.59)	55.66	198	< 0.001
**Type of care**							
Critical care	47	29.53 (9.33)	34.87 (8.40)	5.34 (6.96, 5.53)	3.72	46	< 0.001
Special care	75	36.27(9.68)	42.68 (9.71)	6.41 (5.21, 7.61)	10.66	74	< 0.001
Acute care	391	36.66 (10.41)	42.76 (10.51)	6.10 (5.54, 6.67)	21.21	390	< 0.001

Note: CI = Confidence Interval; LL = Lower Limit; UL = Upper Limit

Table 4 shows the results of Paired samples T test for children in acute care when different caregivers provided them intervention. Paired samples T test was not used for critical care and special care due to small sample size. When mothers gave intervention, significant difference in the scores of MSE-S before and after the intervention was observed (mean difference = 5.87; t(268) = 17.43, *p* < 0.001). Significant difference was observed on the MSE-S before and after the intervention, when intervention was offered by fathers (mean difference = 6.12; t(57) = 7.63, *p* < 0.001). Similarly, a significant difference was observed when intervention was given by other family members on MSE-S before and after the intervention(mean difference = 4.89); t [33] = 5.68, *p* < 0.001. Difference was also observed on the MSE-S before and after the intervention when intervention was given by trainees (mean difference = 9.69; t [27] = 8.74, *p* < 0.001).

**Table 4:** Paired Samples T test of MSE of children based on the presence of attendants in acute care

Domain	*N*	Before Mean (SD)	After Mean (SD)	Mean Difference (95% CI = LL, UL)	*t*	df	Sig
**Attendant present during the session**							
Mother	269	36.14 (10.49)	42.02 (10.68)	5.87 (5.21, 6.54)	17.43	268	< 0.001
Father	58	37.45 (11.64)	43.57 (11.39)	6.12 (4.51, 7.73)	7.63	57	< 0.001
Other caregivers (siblings, grandparents, uncles and aunts)	35	39 (9.16)	43.89 (8.51)	4.89 (3.14, 6.63)	5.68	34	< 0.001
Trainees	29	37.03 (8.26)	46.72 (8.42)	9.69 (5.96, 7.42)	8.74	28	< 0.001

Note: CI = Confidence Interval; LL = Lower Limit; UL = Upper Limit

## Discussion

There were three objectives of the study:(i) to determine if a play stimulation intervention mediated by non-specialist providers improves the mental status of children who are hospitalized; (ii) to examine if there is a difference between parent-mediated and other caregiver-mediated sessions on the child’s MSE pre and post intervention; (iii) to examine if there was variation in the difference by child age and type of care (acute, special and critical).

There was a significant difference in overall score on MSE-S, pre and post interventions. The difference in the score on MSE-S was the greatest when trainees offered interventions as compared to parents. This could be due to professional training in clinical psychology. Students with a background of clinical psychology use the core skills of empathy, unconditional positive regard, genuineness, and active listening. They are well versed in developmental milestones as well as different theories of development, hence they can help children in a better way as compared to the parents who are not offered such training or seek such training. Another reason could be parental stress and the changes in responsiveness [25]. This new role of taking care of a sick child in a hospital setting can be perceived by parents as challenging and destabilizing [26]. Parents, especially mothers face challenges such as vulnerabilities at individual and household facility levels. It is also perceived that mothers are blamed for the sickness of their children, which adds to their stress [27]. Fathers are less likely to use the support given to them from the hospital [28]. Hence, such challenges can impact the interaction of parents and children in a hospital environment.

Significant difference was also observed in the scores of MSE-S when both mothers and fathers offered intervention. The score of mental status of children was the same when both mothers and fathers gave the intervention. Similar score in parental response could be because of the trainees teaching responsive care to caregivers [19]. Parents feel supported and confident when support is provided to them by professionals [29]. In responsive caregiving, parents talk to the staff about stress which then helps in reducing the stress [30]. Parents who are involved in childcare during hopsitalization are better able to cope with their role, hence it can influence responsive caregiving [31]. Parent supportiveness helps in reducing a child’s negative behaviour [32].

Another observation was that children responded well to the fathers which was surprising since Pakistan is a patriarchal society and mothers are involved in child rearing [33]. Fathers play an important role in the upbringing of children (35). Father-child relationship is dependent on the quantity and quality of father-child behaviour. When this remains relatively stable across childhood, there is increased paternal sensitivity overtime [25].

One finding was that there was a change in the scores of MSE-S when the intervention was offered by parents and as well as other caregivers. Children with illnesses benefit from close connection with family. Studies have suggested that support from parents and siblings lead towards resilience in the family as well as the child when there are medical procedures during hospitalization [34]. Parental distress and children’s own distress changes when they have access to recreational rooms in hospital settings [35]. The mutual joy and communication that the parents and children share reduces the body’s stress response [36]. Play specialist-based interventions have been found to reduce anxiety in both parents and children after the surgery of their children [37]. Parents highly recommend that psychosocial stimulation for hospitalized children aged 3 to 6 years is helpful and leads towards better health outcomes for children [38, 39]. Since some caregivers in the intervention, were grandparents too, children responded well to them. Grandmothers are emotionally closer to children and are more involved in caretaking [40]. Children are more comfortable with their parents and other caregivers in a hospital environment and they also respond well to trainees as they employ the core psychotherapeutic skills during their interactions.

Other findings include the significant difference on the score of MSE-S before and after the intervention. It can be deduced that psychosocial stimulation not only impacts the mood of children but also the cognitive functioning and motor skills as can be observed by the increase in score on MSE-S. Play enhances brain functioning by using the executive function. Children with life threatening conditions face obstacles that negatively impact development. Play induces cognitive, emotional, social and psychomotor functioning [11]. Parent-mediated interventions based on early child development practices have a positive impact on cognitive functioning right after the intervention [33]. Interventions given by parents have a positive effect on cognition, language, motor development and parent child interactions [33, 41]. Parent-mediated interventions lead to responsive caregiving during and after hospitalization [19]. Therefore, parent-mediated interventions can have a positive impact on behavioural and cognitive functioning of children which are a part of mental status.

Greatest difference before and after the intervention was observed between children aged 24 months to 60 months. Play-based interventions reduce stress related to medical procedures like needle-related medical procedures in toddlers [42]. Anxiety in hospitalized preschoolers decreases after play therapy interventions are given to them such as colouring [43, 44]. Play plays a vital role in the healthy development of children. It leads to physical, emotional and cognitive benefits [11].

Interventions also improved the mental status of children in different types of care such critical care, special care and acute care units. Interventions like painting for preschoolers in intensive care units help in projecting and releasing emotions along with reducing stress and cooperating with health care providers [45, 46]. For chronically ill children, healthy play helps in promoting psychosocial, social, cognitive and psychomotor functioning [11]. Though there are not several studies on the impact of play stimulation for hospitalized children admitted in intensive and special care units, several studies have lighted the importance of incorporating psychosocial care in intensive care [47, 49].

The study is not without limitations. Though the findings in the study have been based on the observations of the trainees, response bias is something that cannot be ignored. There is a possibility that the trainees may have responded pertaining to their own subjective experience rather than objective experience. Studies in hospital can be biased such that some consultants can be more supportive hence more patients are referred by them because of which majority of the patients can be from one section such as cardiology or infectious diseases. Another limitation is that the observation scales have not been validated as there was not any simultaneous rating of one patient by two trainees, the findings have to be considered with caution. There are several variables that can impact the scores on the observation forms. Such variables may be in terms of the environment, the mental status of the caregivers, as well as children’s own history of previous admissions. Such variables can be considered as another study that impact the mental status of children and as well as their interactions with others. Another limitation could be the design of the study which is a one group pre and posttest design. An experimental design with a control group which would further help in understanding whether the changes observed were specifically through intervention or other extraneous variables.

Overall, it can be concluded that offering play stimulation not only impacts the behaviour of children but also the response of caregivers to the children. Play stimulation that is offered by trainees and parents show a significant difference in the mental status of children after the intervention. Mothers and fathers both feel stressed out. At times there can be other determinants apart from the caregivers response that can determine the mood of children while hospitalized. Individuals trained in psychosocial stimulation can also influence the mental status of children. The study gives an overall outlook towards the relationship of children and caregivers during hospitalization as well as the influence of play stimulation on the mental status of children in the context of a private hospital setting in Pakistan.

### Electronic supplementary material

Below is the link to the electronic supplementary material.

Supplementary Material 1

## Data Availability

Some of the materials used to conduct the study are presented in a public archive: https://www.sciencedirect.com/science/article/pii/S2666560322000664.

## Code availability

Analytic code used to conduct the analyses presented in this study are not available in a public archive. They may be available by emailing the principal investigator at muneera.rasheed21@gmail.com.

## Data availability

Data is available upon request. It may be available by emailing the principal investigator at muneera.rasheed21@gmail.com.

**Study registration**: this study was not formally registered. **Analytic plan pre-registration**: the analysis plan was not formally pre-registered. De-identified data from this study are not available in a public archive. De-identified data from this study will be made available (as allowable according to institutional IRB standards) by emailing the principal investigator of the study.
