# Supplementary material for: Effect of play-based intervention on children’s mental status and caregiver involvement during hospitalization: findings from Pakistan
Source: BMC Pediatr. 2024 Apr 4;24:239. doi: 10.1186/s12887-024-04659-5 (PMC10993442; doi:10.1186/s12887-024-04659-5)
Supplement: Supplementary file 1 — Supplementary Material 1 [file 12887_2024_4659_MOESM1_ESM.docx]

Supplementary Table 1: Independent samples T test of behavioral observation of parents and children.

| Domain | Mother  N, M (SD) | Father  N, M (SD) | Mean Difference | t (p-value) |
| --- | --- | --- | --- | --- |
| Family Positive Affect | 163, 10.20 (2.14) | 44, 10.57 (1.77) | -0.37 | -1.04 (0.300) |
| Family Behaviour Attunement | 163, 8.09 (2.67) | 43, 8.16 (2.69) | -0.07 | -0.15 (0.516) |
| Family Communication | 125, 10.35 (3.55) | 35, 11.06 (3.24) | -0.71 | -1.06 (0.292) |
| Family Stress | 154, 9.88 (2.56) | 43, 10.67 (1.94) | -0.79 | -2.20 (0.031) |
| Children Behaviour Attunement | 86, 11.63 (3.71) | 22, 9.55 (4.90) | 2.08 | 1.86 (0.073) |
| Children Language | 105, 7.24 (1.55) | 23, 6.61 (2.04) | -2.21 | 1.39 (0.174) |
| Children Positive Affect | 91, 6.51 (3.86) | 21, 5.29 (4.09) | 1.22 | 1.29 (0.199) |
| Overall Family Observation | 170, 43.77 (10.79) | 46, 45.98 (10.82) | 4.49 | -1.23 (0.220) |
| Overall Children Observation | 61, 45.11 (11.25) | 15, 37.93 (12.92) | 7.18 | 2.15 (0.035) |
